# A DedA Family Membrane Protein Is Required for *Burkholderia thailandensis* Colistin Resistance

**DOI:** 10.3389/fmicb.2019.02532

**Published:** 2019-11-05

**Authors:** Pradip R. Panta, Sujeet Kumar, Caroline F. Stafford, Caitlin E. Billiot, Martin V. Douglass, Carmen M. Herrera, M. Stephen Trent, William T. Doerrler

**Affiliations:** ^1^Department of Biological Sciences, Louisiana State University, Baton Rouge, LA, United States; ^2^Department of Infectious Diseases, University of Georgia College of Veterinary Medicine, Athens, GA, United States; ^3^Center for Vaccines and Immunology, University of Georgia College of Veterinary Medicine, Athens, GA, United States

**Keywords:** colistin, antibiotic resistance, lipopolysaccharide, membrane protein, proton motive force

## Abstract

Colistin is a “last resort” antibiotic for treatment of infections caused by some multidrug resistant Gram-negative bacterial pathogens. Resistance to colistin varies between bacterial species. Some Gram-negative bacteria such as *Burkholderia* spp. are intrinsically resistant to very high levels of colistin with minimal inhibitory concentrations (MIC) often above 0.5 mg/ml. We have previously shown DedA family proteins YqjA and YghB are conserved membrane transporters required for alkaline tolerance and resistance to several classes of dyes and antibiotics in *Escherichia coli*. Here, we show that a DedA family protein in *Burkholderia thailandensis* (DbcA; DedA of *Burkholderia* required for colistin resistance) is a membrane transporter required for resistance to colistin. Mutation of *dbcA* results in >100-fold greater sensitivity to colistin. Colistin resistance is often conferred via covalent modification of lipopolysaccharide (LPS) lipid A. Mass spectrometry of lipid A of Δ*dbcA* showed a sharp reduction of aminoarabinose in lipid A compared to wild type. Complementation of colistin sensitivity of *B. thailandensis* Δ*dbcA* was observed by expression of *dbcA*, *E. coli yghB* or *E. coli yqjA*. Many proton-dependent transporters possess charged amino acids in transmembrane domains that take part in the transport mechanism and are essential for function. Site directed mutagenesis of conserved and predicted membrane embedded charged amino acids suggest that DbcA functions as a proton-dependent transporter. Direct measurement of membrane potential shows that *B. thailandensis* Δ*dbcA* is partially depolarized suggesting that loss of protonmotive force can lead to alterations in LPS structure and severe colistin sensitivity in this species.

## Introduction

Colistin (polymyxin E) is a last resort antibiotic for treatment of infections caused by Gram-negative pathogenic bacteria (Paterson and Harris, 2016). Discovered in 1947 (Ainsworth et al., 1947), polymyxins were rarely used internally due to nephrotoxicity (Koch-Weser et al., 1970). However, their use has increased recently due to ineffectiveness of approved antibiotics against multidrug-resistant bacteria, including carbapenemase-producing *Enterobacteriaceae* such as *Klebsiella pneumoniae* (Queenan and Bush, 2007). However, many Gram-negative bacteria are intrinsically resistant to colistin and plasmid-acquired resistance has recently been reported (Liu et al., 2016; McGann et al., 2016; Rolain et al., 2016; Sun et al., 2018).

The genus *Burkholderia* is a group of highly adaptable, Gram-negative bacteria that includes a number of animal and plant pathogens (Lipuma, 2010; Waters, 2012). In terms of human infections, *Burkholderia* is extremely difficult to treat due to resistance to a number of commonly used antibiotics, arising from the presence of numerous multidrug resistance efflux pumps as well as restricted permeation (Podnecky et al., 2015; Krishnamoorthy et al., 2017). *Burkholderia* spp. exhibit extremely high intrinsic polymyxin resistance with minimal inhibitory concentrations (MIC) often exceeding 500 μg/ml (Loutet and Valvano, 2011), two orders of magnitude greater than that observed for most Gram-negative species. *Burkholderia thailandensis* is closely related to *Burkholderia pseudomallei*, the cause of melioidosis (Wiersinga et al., 2018), and both species are highly resistant to colistin and most other antibiotics (Krishnamoorthy et al., 2019). While *B. thailandensis* is considered a suitable surrogate for *B. pseudomallei* and only rarely causes infections in humans, it is infectious in a number of mammalian tissue culture, murine, insect and plant models and possesses virulence factors and drug resistance mechanisms that are found in its more virulent relatives (Gallagher et al., 2013). The existence of an ordered transposon library also makes *B. thailandensis* a valuable model organism to study *Burkholderia* virulence (Gallagher et al., 2013).

Cationic peptides produced by the immune system and antibiotics such as colistin interact electrostatically with negatively charged outer membrane of Gram negative bacteria. A common mechanism that *Burkholderia* spp. share with other Gram-negative species involves expression of a biosynthetic pathway that results in the modification of LPS lipid A with aminoarabinose (Ara4N). This amine-containing group neutralizes the negative charge of lipid A inhibiting colistin binding (Simpson and Trent, 2019). The remarkably high colistin resistance of *Burkholderia* spp. is likely due to a combination of a number of factors such as LPS modifications, as well as hopanoid synthesis (Malott et al., 2012), secreted metalloproteases (Loutet and Valvano, 2011) and synthesis of antioxidant putrescine (El-Halfawy and Valvano, 2014).

The DedA/Tvp38 membrane protein family (DedA family for short) is a highly conserved protein family that remains poorly characterized. There are currently 27,035 individual sequences in the protein database across 8547 species belonging to the “SNARE-associated PF09335” family of proteins (PFAM 31.0). We have characterized members of the DedA family in *Escherichia coli* and *Borrelia burgdorferi* (Thompkins et al., 2008; Liang et al., 2010; Sikdar and Doerrler, 2010; Boughner and Doerrler, 2012; Doerrler et al., 2013; Sikdar et al., 2013; Kumar and Doerrler, 2014, 2015; Kumar et al., 2016). The DedA family includes *E. coli* YqjA and YghB; putative proton dependent transporters that together are required for normal growth and cell division (Thompkins et al., 2008; Sikdar and Doerrler, 2010) and resistance to a number of antibiotics and biocides (Kumar and Doerrler, 2014) while YqjA is alone required for alkaline tolerance (Kumar and Doerrler, 2015). Both YqjA and YghB possess essential membrane embedded charged amino acids (Kumar and Doerrler, 2014; Kumar et al., 2016) that are present in proton-dependent transporters belonging to the major facilitator superfamily and other families (Noumi et al., 1997; Abramson et al., 2004; Adler and Bibi, 2004; Sigal et al., 2005; Fluman et al., 2012; Holdsworth and Law, 2012). While the reasons for this are unclear, DedA family proteins are required for polymyxin and/or antimicrobial peptide resistance of *Salmonella enterica* (Shi et al., 2004), *Neisseria meningitidis* (Tzeng et al., 2005), *E. coli* (Weatherspoon-Griffin et al., 2011), *K. pneumoniae* (Jana et al., 2017) and *Enterobacter cloacae* (Huang et al., 2019).

The energetic requirements of lipid A modification required for colistin resistance are not well characterized. Periplasmic modification of lipid A by ArnT requires cytoplasmic synthesis and inner membrane transport of undecaprenyl-P-Ara4N (Simpson and Trent, 2019). The transport of this lipid-linked intermediate in *E. coli* is carried out by membrane transporters designated ArnE and ArnF which are similar to small multidrug resistance protein EmrE (Yan et al., 2007). Dephosphorylation and (possibly) recycling of undecaprenyl pyrophosphate is carried out by the membrane protein UppP/BacA that displays low similarity to major facilitator superfamily transporter MdfA and other transporters (El Ghachi et al., 2018; Workman et al., 2018). We have previously demonstrated that proton motive force (PMF)-dependent resistance to EmrE and MdfA substrates such as methyl viologen and ethidium bromide is compromised in an *E. coli dedA* family mutant (3). Our hypothesis is that transport and/or recycling of undecaprenyl-P-Ara4N and undecaprenyl-P, respectively, is inefficient in DedA family mutants resulting in production of lipid A with lower amounts of Ara4N. In this work, we characterized a *B. thailandensis* DedA family mutant and observed altered lipid A with reduced amounts of Ara4N compared to the parent strain. In addition, we demonstrate the essentiality of membrane embedded charged amino acids in the DedA protein for colistin resistance. Further, we show that membrane potential is reduced in this mutant and membrane potential is itself required for colistin resistance. These results collectively suggest that the transport activity of DedA and tight control of PMF is required for lipid A modification with Ara4N and colistin resistance of *B. thailandensis*.

## Materials and Methods

### Culture Conditions

*Burkholderia thailandensis* E264 and transposon mutants disrupting the *dbcA* gene were acquired from the Manoil lab (University of Washington)^1^ (Gallagher et al., 2013). *E. coli* cultures were grown in lysogeny broth (LB) medium (1% tryptone, 0.5% yeast extract and 1% NaCl). *B. thailandensis* was grown in LB or cation adjusted Mueller-Hinton broth 2 (MH2; typical final pH 7.3. Sigma-Aldrich). Antibiotics colistin, ampicillin (Amp) 100 μg/ml, kanamycin (Kan) 30 μg/ml (*E. coli*) or 100 μg/ml (*B. thailandensis*), tetracycline (Tet) 12.5 μg/ml, and trimethoprim (Tmp) 100 μg/ml were purchased from Sigma-Aldrich or VWR. Cultures were grown at 37°C unless otherwise indicated.

### Deletion of *B. thailandensis* E264 *dbcA*

For targeted mutagenesis of *Bth_I1321* (herein referred to as *dbcA*; NCBI GenBank: ABC36705.1), we used natural transformation of PCR fragments (Thongdee et al., 2008). All oligonucleotides used in PCR were purchased from Sigma-Aldrich and are listed in Supplementary Table S2. Using P1F and P1R, a 1064 bp upstream region of *DbcA* was PCR-amplified using Q5 DNA polymerase (New England BioLabs). Primers P2F and P2R were used to amplify 980 bp downstream of *dbcA* (Supplementary Table S2). DNA elements carrying the *dhfrIIa* gene encoding for trimethoprim resistance (Tmp^R^) was PCR amplified from pUC18T-mini-Tn7T-Tmp plasmid using TmpF and TmpR primers. The plasmid, pUC18T-mini-Tn7T-Tmp was a gift from Dr. Colin Manoil (University of Washington). Primers P1R and TmpF have 40 bp overlapping ends and so do primers P2F and TmpR as shown in Supplementary Table S2 (underlined text). All three amplified fragments were ligated in one step using the Gibson Assembly kit (New England Biolabs). One microliter of the ligated product was used as a template for PCR amplification using P1F and P2R primers. The amplified product (2986 bp) was confirmed by 1% agarose gel electrophoresis and purified using QIAquick Gel Extraction Kit (Qiagen).

For transformation of *B. thailandensis* E264 with the purified PCR product, a defined medium (DM) was made consisting of 0.25X M63 supplemented with 0.2% glucose, 0.4% glycerol, 1 mM MgSO_4_, 1 μg/ml thiamine, and six amino acids- leucine, isoleucine, valine, tryptophan, glutamic acid, and glutamine (40 μg/ml each) (19). E264 was grown overnight in LB. The culture was then diluted 1:100 (∼50 μl) in 5 ml of DM media and grown with shaking at 37°C until an optical density at 600 nm of ∼0.6 was reached. 1 ml of culture was centrifuged for 1 min and resuspended in 200 μl of DM. 50 μl aliquots of resuspended cells were mixed with either 0, 100, or 200 ng of the PCR product in a 5 μl volume. The mixtures were incubated without agitation for 30 min at room temperature, and then 1 ml of DM was added and incubated for more than 6 h with shaking at 37°C. The cells were washed twice with 1 ml DM, then resuspended in 250 μl of DM. Different dilutions were made and plated on LB supplemented with Tmp 100 μg/ml to select for recombinants and incubated at 37°C. Genomic DNA was extracted from Tmp^R^ clones using Easy-DNA kit (Invitrogen). Genomic DNA was used as a template and primers – Confirm FW and Confirm REV were used to confirm the recombinants. Primers- Seq FW and Seq REV were used to amplify the region, and it was cloned into pBBR1MCS-2 (Kovach et al., 1995) in *Xho*I and *Hin*dIII sites and plasmid specific primers M13FW and M13REV were used to sequence the entire *Xho*I/*Hin*dIII fragment. The Δ*dbcA:*Tmp^R^ replacement was confirmed by DNA Sequencing at the LSU Genomic facility. Removal of the trimethoprim-resistance cassette from Tmp^R^ clones was carried out using pFlpTet (Garcia et al., 2013) leaving an FRT scar (Δ*dbcA:FRT*). The plasmid pFlpTet was a gift from Dr. Erin C. Garcia (University of Kentucky College of Medicine) and was cured at 39°C prior to mutant selection. Some experiments used the strain Δ*dbcA:*Tmp^R^ while others utilized Δ*dbcA:FRT* and the strain used is indicated in the respective figure legends. The Δ*dbcA:*Tmp^S^ clone was sequenced and confirmed as described above. Genomic DNA was extracted from WT, Tmp^R^, and Tmp^S^ clones using Easy-DNA kit (Invitrogen). Genomic DNA was used as a template and primers Seq FW and Seq REV were used to confirm the recombinants (Supplementary Figure S1).

**FIGURE 1:**
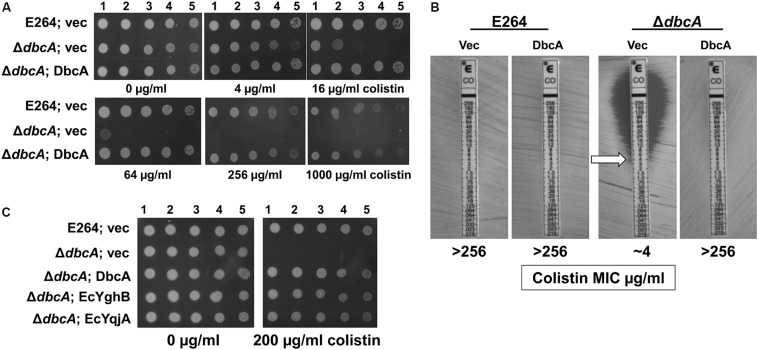
Sensitivity of *Burkholderia thailandensis* E264 Δ*dbcA* to colistin. **(A)** 1:10 dilutions of indicated log-phase grown strains were spotted and grown on MH2 growth medium containing 100 μg/ml kanamycin and either 0, 4, 16, 64, 256, or 1000 μg/ml of colistin. **(B)** MIC was determined for indicated strains using colistin *E*-test strips (Biomerieux). Arrow indicates approximate MIC. **(C)** Δ*dbcA* was transformed with control vector (vec) or pRP101 (*dbcA*), pRP102 (Ec*yqjA*) or pRP103 (Ec*yghB*) directing constitutive expression of the indicated genes and log phase cells were diluted and spotted onto plates containing 100 μg/ml kanamycin and either 0 or 200 μg/ml of colistin. Plates were incubated 24 h at 37°C.

**FIGURE 2:**
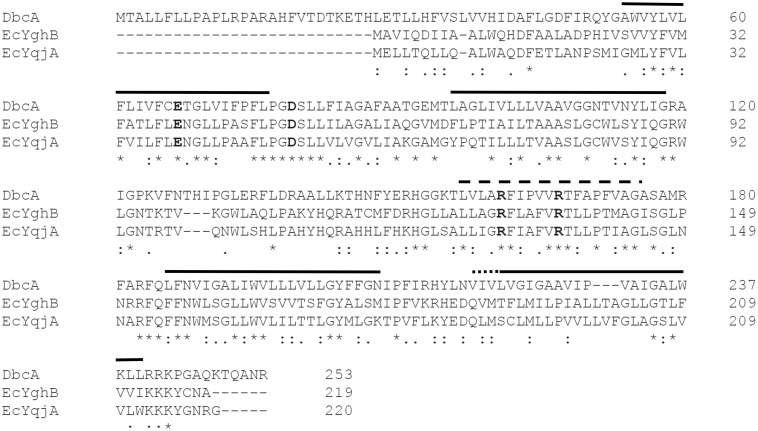
Amino acid similarity between *Escherichia coli* and *B. thailandensis* DedA family proteins. Amino acid alignment of DbcA, EcYghB and EcYqjA using Clustal Omega (Sievers and Higgins, 2014). Functional acidic (E39, D51; YghB numbering) and basic (R130, R136; YghB numbering) amino acids are in bold font (Kumar and Doerrler, 2014; Kumar et al., 2016). Solid lines represent predicted transmembrane helices of DbcA and EcYghB using TMHMM (Krogh et al., 2001). Residues 156–175 (dashed line) are hydrophobic in nature but the software does not predict a true transmembrane helix. This region may instead be a “re-entrant” helix, similar to what was observed in the crystal structures of UppP/BacA (El Ghachi et al., 2018; Workman et al., 2018). Residues 218–220 of DbcA (dotted line) do not align with the beginning of the same helix of YghB. An asterisk indicates positions that have a single, fully conserved residue. A colon indicates conservation between strongly similar amino acids. A period indicates conservation between weakly similar amino acids.

### Site-Directed Mutagenesis

For making point mutants in *dbcA* gene, we cloned the *dbcA* gene under the inducible rhamnose promoter in pSCrhaB2 vector (Cardona and Valvano, 2005) using primers FWdbcAhis and REVdbcAhis in such a way to add a hexahistidine tag at the C terminus (Iiyama et al., 2011). The plasmid pScrhaB2 was a generous gift of Dr. Josephine Chandler (University of Kansas, Dept. of Molecular Biosciences). Site-specific mutations were created according to a previously published protocol (Kumar and Doerrler, 2014). The primers with site specific mutations (Supplementary Table S2) were used to amplify the entire vector containing the *dbcA* gene. The PCR products were digested with *Dpn*I, purified with a Qiagen kit and used to transform competent XL1-Blue cells. Transformants were screened by gene specific primers. Point mutations were confirmed by DNA sequencing at the LSU College of Science Genomic Facility. Membrane preparation and Western blotting using an anti-pentahis antibody (Qiagen) was performed as previously described (Kumar and Doerrler, 2014).

### Transformation and Complementation Analysis

For transformation of *E. coli*, a common heat shock method was used (Froger and Hall, 2007). For *B. thailandensis*, washes and electroporation was carried out at room temperature, which improved transformation efficiency (Tu et al., 2016). *B. thailandensis* was grown in LB with shaking at 37°C until an optical density at 600 nm of ∼0.6 was reached. The cells were washed once with water and twice with 10% glycerol. The cells were resuspended with ∼300 μl of 10% glycerol to a concentration 2–3^∗^10^10^ cells/ml. A 50 μl aliquot was mixed with ∼500 ng of plasmid DNA and electroporated using a Bio-Rad MicroPulser using a 0.2 cm cuvette and 2.5 kV voltage setting. One ml of warm SOC media was added immediately after the pulse and incubated for 1.5 h with shaking at 37°C. The cells were centrifuged and resuspended in 100 μl of SOC, plated on LB plates with appropriate antibiotics, and incubated at 37°C for up to 48 h.

For complementation experiments, all genes were cloned under a constitutive *lac* promoter of an expression vector, pBBR1MCS-2 (Kovach et al., 1995) and strains selected in the presence of Kan 100 μg/ml (*B. thailandensis*) or 30 μg/ml (*E. coli*). The unmodified plasmid was used as vector control in all experiments. *E. coli* DedA genes *yqjA (EcyqjA*) and *yghB* (*EcyghB*) were cut from pBAD-*yqjA* and pBAD-*yghB* (Sikdar et al., 2013) with *Xho*I and *Hin*dIII and ligated into a similarly digested pBBR1MCS-2 vector using T4 DNA ligase resulting in pRP102 and pRP103, respectively. *B. thailandensis DbcA* was PCR amplified from genomic DNA of *B. thailandensis* using FWdbcA and REVdbcA primers and ligated into *Xho*I and *Hin*dIII sites of pBBR1MCS-2 resulting in pRP101. For construction of kanamycin-sensitive strain BC202KS, the kanamycin resistance cassette of BC204 (Δ*yghB*:Kan^R^) (Thompkins et al., 2008) was removed using Flp recombinase expressed from plasmid pCP20 (Cherepanov and Wackernagel, 1995; Datsenko and Wanner, 2000; Boughner and Doerrler, 2012). Following curing of pCP20 at 42°C, the resulting strain was transduced to tetracycline resistance using a p1vir lysate prepared from strain BC203 (Δ*yqjA*:Tet^R^) (Thompkins et al., 2008), resulting in kan-sensitive BC202KS (Supplementary Table S1).

### Microscopy

Overnight cultures of *E. coli* were diluted 1:100 in fresh LB media with suitable antibiotics and additives, and grown to OD_600_ ∼ 0.6 at 30°C in a shaking incubator. Ten μL of cells were applied to a 1% agarose coated glass slide for imaging. A Leica DM6B-Z deconvolution microscope was used for all the differential interference contrast (DIC) micrographs. Observations were made by a 100X, 1.44-numerical-aperture oil immersion objective lens (HC PL APO). The images were captured through Hamamatsu C11440-22C, 16 bit camera and recorded using Leica Application Suite X (LAS X) software.

### Susceptibility to Colistin and Other Antibiotics

For testing the susceptibility on solid medium, overnight cultures were freshly diluted 1:100 in LB or MH2 media with appropriate antibiotics and additives, and grown to OD_600_ ∼0.6 at 37°C in a shaking incubator. Five microliters of serially log_10_-diluted cells were spotted onto MH2 or LB agar plates containing antibiotics. Growth was analyzed after incubation for 24 h at 37°C. The MIC was measured with colistin *E*-test strips (Biomerieux). Overnight cultures were diluted 1:100 into fresh MH2 broth and grown to OD_600_ 0.6. A 1:10 dilution was spread on MH2 plates to create a lawn of cells and the strip was applied to the plates and evaluated after 24 h at 37°C. All experiments were repeated at least three times.

### Mass Spectrometry

For isolation of lipid A, cultures were grown at 37°C to an OD_600_ of ∼1.0. Lipid A chemical extraction was carried out after mild acidic hydrolysis of LPS as previously described (Zhou et al., 1999; Herrera et al., 2014). For visualization of lipid A by mass spectrometry, lipids were analyzed using MALDI-TOF (ABI 4700 Proteomic Analyzer) in the negative-ion linear mode as previously described (Zhou et al., 2010; Henderson et al., 2013). Briefly, lipid A samples were dissolved in a mixture of chloroform-methanol (4:1, vol/vol), and 1 μl of sample was mixed with 1 μl of matrix solution. The matrix consisted of 5-chloro-2-mercaptobenzothiazole (CMBT) (20 mg/mL) resuspended in chloroform-methanol-water (4:4:1, vol/vol/vol) mixed with saturated ammonium citrate (20:1, vol/vol). One μl of sample-matrix mixture was loaded on to MALDI target plate for final analysis.

### Determination of Membrane Potential

Assessment of membrane potential (Δψ) using JC-1 dye was as described (Sikdar et al., 2013). Briefly, All strains were treated with 4 μM JC-1 dye in permeabilization buffer (10 mM Tris, pH 7.5, 1 mM EDTA, 10 mM glucose), incubated in the dark at 30°C without shaking for 30 min and fluorescence measurements carried out using a JASCO FP-6300 spectrofluorometer. Strains treated with CCCP for 30 min served as a control for the loss of Δψ.

## Results

### Colistin Sensitivity of the *B. thailandensis*Δ*dbcA*

By screening several sequence-defined *B. thailandensis* transposon mutants obtained from the Manoil lab (Gallagher et al., 2013) in genes encoding DedA family members we discovered that several with confirmed insertions in the gene *Bth_I1321* (referred to herein as *dbcA*; DedA of *Burkholderia* required for colistin resistance) were highly sensitive to colistin and polymyxin B. To confirm this observation in a gene deletion strain, we created a *B. thailandensis* strain where the *dbcA* locus was replaced with a trimethoprim resistance (Tmp^R^) gene (*dhfrIIa*). Gene replacement and removal of the Tmp^R^ cassette in strain Δ*dbcA* was confirmed by PCR and DNA sequencing (Supplementary Figure S1). While wild type strain E264 and complemented Δ*dbcA* grew in the presence of high concentrations of colistin (>1000 μg/ml), Δ*dbcA* carrying the control vector failed to grow at colistin concentrations greater than about 4 μg/ml (Figure 1). This was shown by spotting dilutions of log-phase cells (Figure 1A) and by using colistin *E*-test gradient strips (Figure 1B). Expression of *E. coli yghB* (*EcyghB*) or Ec*yqjA* could restore growth of Δ*dbcA* in the presence of high concentrations of colistin (Figure 1C). DbcA displays moderate amino acid identity to EcYqjA (∼34%) and EcYghB (∼30%) (Figure 2).

### Mass Spectrometry Analysis Reveals Reduced Levels of Ara4N-Modified Lipid A in Δ*dbcA* Mutant

**FIGURE 3:**
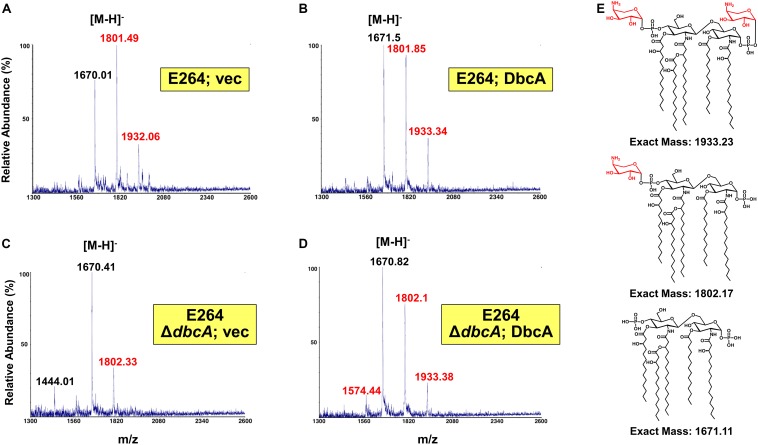
Mass spectrometry of lipid A isolated from *B. thailandensis* strains. **(A–D)** Lipid A extracted from the indicated strains was analyzed using a MALDI-TOF mass spectrometer (ABI 4700 Proteomic Analyzer) in the negative-ion linear mode. Species modified with Ara4N are labeled in red font. **(E)** Predicted structures of each observed species. Minor species of unmodified tetraacylated lipid A (m/z = 1444.01) and singly Ara4N-modified tetraacylated lipid A (m/z = 1574.44) were also detected.

Since lipid A is the initial interaction site of polymyxins, and lipid A modifications contribute to colistin resistance in numerous Gram-negative species, lipid A structure was analyzed by mass spectrometry. In the analysis of wild type *B. thailandensis* E264 harboring control and complementing vector as well as the Δ*dbcA* strain harboring complementing vector, we observed three major peaks (m/z ∼ 1670.01, 1801.49, and 1932.06) which correspond to pentaacylated lipid A, pentaacylated lipid A singly modified with Ara4N and pentaacylated lipid A doubly modified with Ara4N (Figures 3A,B,D). Lipid A species containing aminoarabinose are labeled with red font in Figure 3. While singly modified and unmodified lipid A are major species in these strains, there is a significant amount of doubly modified species as well. Strikingly, in Δ*dbcA* harboring control vector, doubly modified 2-Ara4N lipid A is undetectable and singly and unmodified lipid A are minor and major species, respectively (Figure 3C). There is also a detectable peak at m/z = 1444 which corresponds to unmodified tetraacylated lipid A. The depletion of Ara4N-modified lipid A may contribute to the observed colistin sensitivity in strain Δ*dbcA*. Predicted structures of lipid A species are shown in Figure 3E. Complete absence of Ara4N modified lipid A is not expected in *B. thailandensis* due to the demonstrated essentiality of the glycosyltransferase ArnT in *Burkholderia cenocepacia* (Ortega et al., 2007; Hamad et al., 2012). ArnT is also likely essential in *B. thailandensis* due to the absence of such a transposon mutant in the ordered library (Gallagher et al., 2013) and comparative Tn-Seq analysis (Gislason et al., 2017). Modification of lipid A with phosphoethanolamine was not detected, consistent with the absence of an EptA homolog in the *B. thailandensis* E264 genome (Kim et al., 2005). This observation in combination with the marked sensitivity to colistin of Δ*dbcA* is noteworthy and prompted us to analyze *B. thailandensis* DbcA protein and corresponding Δ*dbcA* strain in more detail.

### Functionality of *B. thailandensis* DbcA in *Escherichia coli*

**FIGURE 4:**
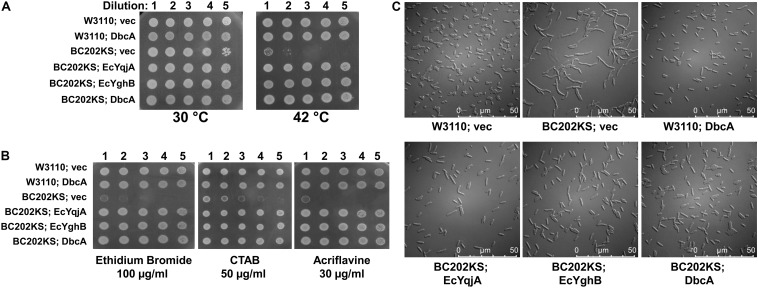
Complementation of *E. coli* BC202 with *dbcA*. **(A)** Restoration of growth of BC202KS at 42°C by plasmid expression of *dbcA*, *EcyqjA* or *EcyghB*. **(B)** Restoration of BC202KS resistance to ethidium bromide, CTAB (cetyltrimethyl ammonium bromide), and acriflavine by plasmid expression of *dbcA*, *EcyqjA* or *EcyghB*. **(C)** Restoration of normal cell division of BC202KS at 30°C by plasmid expression of *dbcA*, *EcyqjA* or *EcyghB*. Abbreviations: Vec, vector control. All strains were grown in LB medium.

The alignment of DbcA with EcYqjA and EcYghB (Figure 2) includes conservation of important membrane embedded acidic (YqjA E39, D51) and basic (YqjA R130, R136) amino acids required for function (Kumar and Doerrler, 2014, 2015; Kumar et al., 2016). To begin to dissect the function of DbcA, we asked whether it could functionally complement altered cell division, temperature sensitivity, and biocide sensitivity of the *E. coli* mutant BC202 (Δ*yqjA*, Δ*yghB*) (Thompkins et al., 2008; Sikdar and Doerrler, 2010; Kumar and Doerrler, 2014). We cloned *dbcA* into pBBR1MCS-2 and the resulting plasmid pRP101 was introduced into parent strain W3110 and mutant BC202KS (a kanamycin-sensitive strain to allow for selection of *Burkholderia* shuttle vectors). pRP102 (Ec*yqjA*) and pRP103 (Ec*yghB*) served as positive controls in these experiments as they can complement these phenotypes of BC202 (Sikdar et al., 2013; Kumar and Doerrler, 2015). As shown in Figure 4, expression of DbcA in BC202KS can restore growth at 42°C (Figure 4A), resistance to biocides (Figure 4B) and normal cell division (Figure 4C) to the same degree as Ec*yqjA* and Ec*yghB*. We conclude that DbcA functions similarly to DedA family proteins YqjA and YghB in *E. coli*.

### Site-Directed Mutagenesis of DbcA Conserved, Charged Amino Acids

**FIGURE 5:**
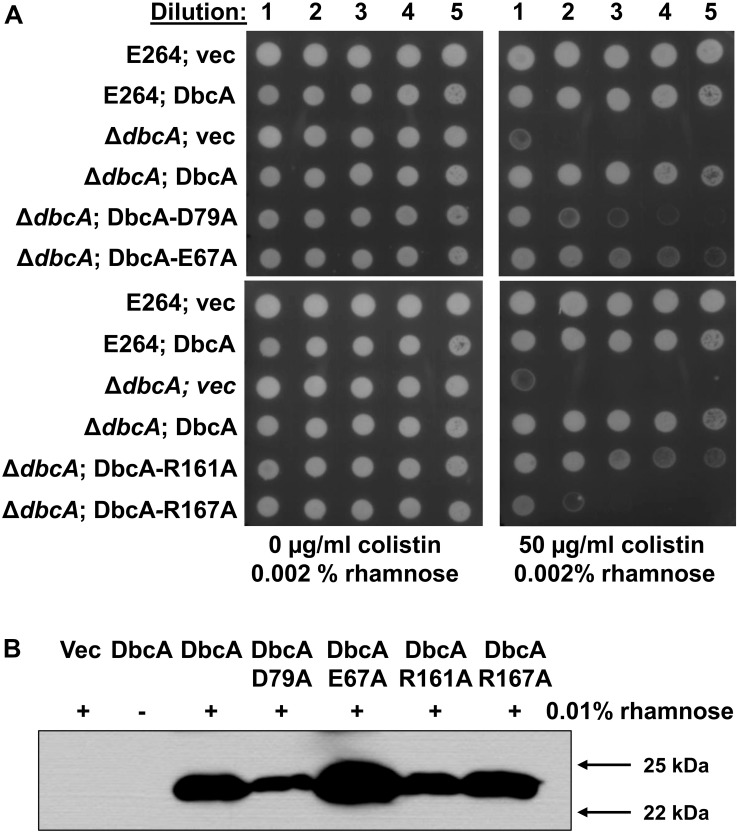
Site directed mutagenesis of *B. thailandensis* DbcA conserved, charged amino acids. **(A)** Dilutions of mid-log phase grown cells of the indicated strains were spotted on MH2 agar plates containing 0.002% rhamnose, 100 μg/ml Tmp and either 0 or 50 μg/ml colistin. This concentration of rhamnose was used to avoid toxicity caused by overexpression of *dbcA* in the wild type strain but was not sufficient for detection using Western blotting (see below). DbcA with mutation D79A or R167A were unable to restore growth in the presence of colistin to the extent of wild type DbcA. **(B)** Expression of *dbcA* and point mutants in membrane fractions of Δ*dbcA* as determined by Western blotting with anti-hexahistidine antibody. All experiments in this figure were conducted with strain Δ*dbcA:FRT* to allow for use of Tmp selection of vectors. Abbreviations: Vec, vector control. Sixty micrograms of membrane protein was loaded per lane and strains were grown in the presence of Tmp and 0.01% rhamnose.

As stated above, DbcA has moderate amino acid identity to the *E. coli* DedA family proteins YqjA and YghB. This conservation includes charged amino acids E67, D79, R161, and R167 that are in comparable positions as YghB/YqjA E39, D51, R130 and R136, respectively (Figure 2), which are required for function (Kumar and Doerrler, 2014; Kumar et al., 2016). Acidic amino acids have been shown to be required for function of numerous secondary transporters including MdfA, NhaA, MdtM, and LacY (Gerchman et al., 1993; Noumi et al., 1997; Abramson et al., 2004; Adler and Bibi, 2004; Sigal et al., 2005; Fluman et al., 2012; Holdsworth and Law, 2012). Furthermore, the significance of membrane embedded basic amino acids such as arginine are well documented in the literature and play a role in a number of processes including regulation of redox potential (Cutler et al., 1989; Winn et al., 2002), voltage detection across a lipid bilayer (Jiang et al., 2003; Long et al., 2005; Tao et al., 2010), and proton transport (Cain and Simoni, 1989; Hellmer et al., 2003; Sigal et al., 2005). We changed each of these amino acids of DbcA to alanine to determine the effect on the ability of the expressed protein to restore colistin resistance to the mutant Δ*dbcA*. All strains grew well in the absence of colistin (Figure 5A). Complementation of growth in the presence of colistin was observed when wild type *dbcA* along with *dbcA*-E67A and *dbcA*-R161A was expressed. Less growth was seen in the presence of colistin when control vector, *dbcA*-D79A or *dbcA*-R167A were expressed. Therefore, we conclude that D79 and R167 likely play major roles in the transport mechanism of DbcA while E67 and R161 may play more minor roles. All proteins were found expressed in the membrane fraction of cells (Figure 5B) suggesting that mutant protein misfolding was not a major issue under our conditions. These results suggest that *B. thailandensis* DbcA functions as a proton-dependent membrane transporter.

### Altered Membrane Potential in Δ*dbcA* Mutant

**FIGURE 6:**
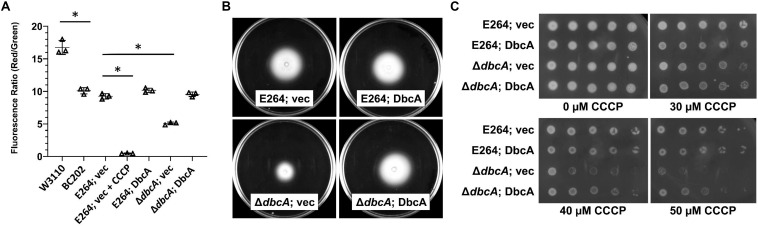
Measurement of membrane potential and CCCP sensitivity. **(A)** Assessment of membrane potential (Δψ) of *B. thailandensis* strains using JC-1 dye represented as the red (595 nm)/green (530 nm) ratio. BC202 was previously shown to be depolarized compared to W3110 (Sikdar et al., 2013) and is included here as a control. E264 (vec) treated with 25 μM CCCP for 30 min served as a control for the loss of Δψ. The graph was drawn using GraphPad Prism 8.1.1 software. Each bar represents the average and standard deviation of three biological replicates. Each experiment was repeated three times. Growth media included 0.0001% rhamnose. **(B)** Impaired motility in Δ*dbcA*:*FRT* strain. For the motility test assay, strains were grown overnight in MH2 and 100 μg/ml Tmp. Five microliters of the overnight culture were inoculated on MH2 media containing 100 μg/ml Tmp and solidified with 0.4% agar and incubated at 37°C for 48 h. No rhamnose was included in the growth media. **(C)** Sensitivity of Δ*dbcA:FRT* to CCCP. Dilutions of strains were spotted on MH2 plates containing 100 μg/ml Tmp, 0.001% rhamnose and the indicated concentration of CCCP and incubated overnight at 37°C. The experiments in panel A-C were conducted with strain Δ*dbcA:FRT* to allow use of Tmp selection (see Supplementary Table S1). Bars represent mean ± SD of three independent determinations and statistical significance was calculated by unpaired Student’s *t*-test using GraphPad Prism 8.1.1. ^∗^*p* < 0.001.

According to chemiosmotic theory (Mitchell, 1961), the membrane PMF is equal to the sum of the charge difference across the membrane (ΔΨ) and the pH difference across the membrane (ΔpH). To examine the PMF in more detail, we measured the ΔΨ component of the PMF using dye JC-1. JC-1 is a membrane permeable dye that exhibits green fluorescence (530 nm) as a monomer but forms aggregates at the membrane in the presence of membrane potential, shifting its emission from green to red (595 nm). Therefore, relative membrane potential can be expressed as the ratio of red to green fluorescence (Jovanovic et al., 2006; Engl et al., 2011). We previously reported that *E. coli* strain BC202 (Δ*yqjA*, Δ*yghB*) displays compromised ΔΨ using this dye (Sikdar et al., 2013). *B. thailandensis*Δ*dbcA* along with wild type harboring either control or complementing vector were grown to mid-log phase and treated with JC-1 dye. Cells treated with the proton ionophore CCCP were included as a control. All wild type and complemented mutant strains exhibited a consistent 595/530 ratio (Figure 6A). However, *B. thailandensis*Δ*dbcA* displayed a lower ratio suggesting partial dissipation of the ΔΨ component of the PMF. This value could alter PMF-dependent processes required for colistin resistance. Non-complemented *B. thailandensis*Δ*dbcA* was also compromised for motility (Figure 6B), and hypersensitive to CCCP (Figure 6C); consistent with partial depolarization of the membrane. This result suggests that loss of PMF can be associated with colistin sensitivity in *B. thailandensis*.

### PMF Depletion Results in Colistin Sensitivity

**FIGURE 7:**
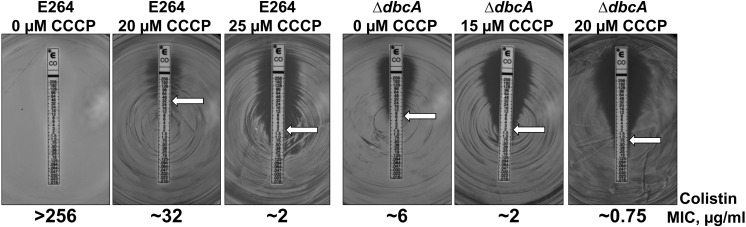
PMF dissipation with CCCP results in *B. thailandensis* colistin sensitivity. Colistin MIC was determined for indicated strains using *E*-test strips (Biomerieux). MH2 plates contained 0, 15, 20 or 25 μM CCCP as indicated and 100 μg/ml Tmp. White arrows indicate approximate MIC.

**FIGURE 8:**
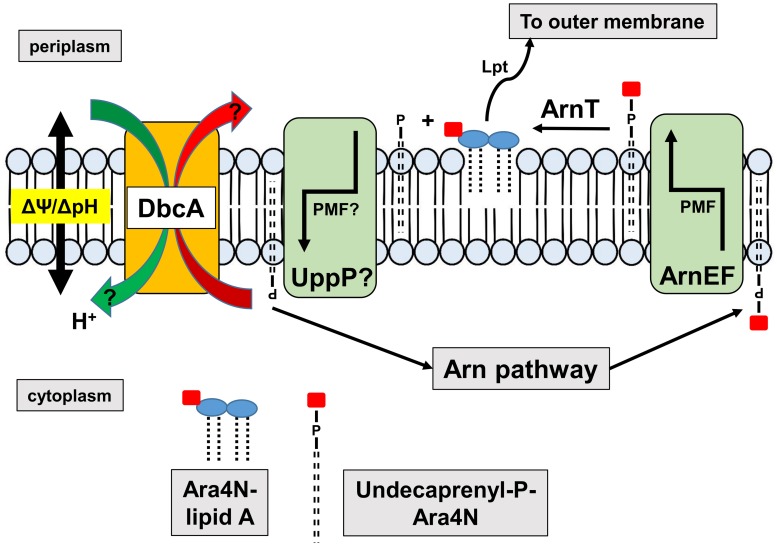
Requirement of DedA family and PMF for efficient synthesis of Ara4N-lipid A. The Arn operon produces undecaprenyl-P-Ara4N that is found initially in the cytoplasmic leaflet of the inner membrane. The EmrE-like transporters ArnEF catalyze the transbilayer movement of undecaprenyl-P-Ara4N to the periplasmic surface of the inner membrane (Yan et al., 2007) where it is a substrate of the glycosyltransferase ArnT (Petrou et al., 2016; Tavares-Carreon et al., 2016). ArnT modifies lipid A with Ara4N on the periplasmic surface of the inner membrane, followed by transport to the outer membrane by the lipopolysaccharide transport pathway (Lpt) (Li et al., 2019; Owens et al., 2019) contributing to colistin resistance. Undecaprenyl-P produced is recycled to the cytoplasm possibly by UppP/BacA, the undecaprenyl pyrophosphate phosphatase, which bears similarity to MdfA and other transporters (El Ghachi et al., 2018; Workman et al., 2018). Both MdfA (Fluman et al., 2012) and EmrE (Robinson et al., 2017) are proton-dependent transporters suggesting that the PMF may be required for both the transbilayer movement of undecaprenyl-P-Ara4N and the recycling of undecaprenyl-P. Perturbation of the PMF in DedA family mutants may interfere with either or both of these steps. We have shown that DedA family proteins are likely proton-dependent transporters and efflux of MdfA and EmrE substrates is compromised in the *E. coli* DedA family mutant BC202, resulting in hypersensitivity to these compounds (Kumar and Doerrler, 2014, 2015).

In order to test if PMF depletion can directly cause sensitivity to colistin, we grew both parent strain *B. thailandensis* E264 and mutant Δ*dbcA* in the presence of CCCP and measured MIC of colistin. We used CCCP concentrations that we determined could reduce the ΔΨ but still allow for survival of the bacteria (Figure 6). As depicted in Figure 7, growth in the presence of CCCP results in significant sensitivity to colistin. The MIC of E264 reduced from unmeasurable to approximately 32 μg/ml or 2 μg/ml in the presence of 20 or 25 μM CCCP, respectively. The colistin MIC of Δ*dbcA* was reduced from 6 μg/ml to approximately 2 μg/ml or 0.75 μg/ml in the presence of 15 or 20 μM CCCP, respectively. This result is consistent with previous findings showing exposure to CCCP sensitizes other species of Gram-negative bacteria to colistin (Park and Ko, 2015; Ni et al., 2016; Osei Sekyere and Amoako, 2017; Baron and Rolain, 2018). These results strongly support our hypothesis of a requirement of PMF for efficient modification of lipid A with Ara4N and colistin resistance in *B. thailandensis* and likely other Gram-negative species (Figure 8). DedA family protein DbcA is required for PMF maintenance in this model.

## Discussion

Multidrug resistant bacterial infections pose an enormous public safety risk and are a challenge to modern medicine, made worse by a lack of new antimicrobial drugs (O’Neill, 2014; World Health Organization [WHO]^∗^, 2014). The emergence of carbapenem-resistant *Enterobacteriaceae* is of particular concern and has forced the reintroduction of colistin therapy as a last resort treatment. Colistin is an antimicrobial peptide belonging to the polymyxin family that can cause numerous side effects including nephrotoxicity (Koch-Weser et al., 1970). Moreover, some epidemic clones of *K. pneumoniae* have acquired colistin resistance by LPS modification via cationic substitution. Other *Enterobacteriaceae* such as *Proteus mirabilis* and *Serratia marcescens* are naturally resistant to polymyxins due to the constitutive expression of the *arnBCADTEF* operon and/or the *eptB* gene (Poirel et al., 2017). The role of the DedA family in providing intrinsic resistance to polymyxins in *Burkholderia* spp. has not been investigated prior to this study. Here we demonstrate that the loss by mutation of DedA homolog DbcA lowers the colistin MIC of *B. thailandensis* to 5–10 μg/ml or at least 100-fold, indicating that this protein plays a key role in determining the resistance phenotype in this species. Although *dedA* family genes have been identified in screens for colistin sensitivity in several species (Shi et al., 2004; Tzeng et al., 2005; Weatherspoon-Griffin et al., 2011; Jana et al., 2017; Huang et al., 2019), this work represents the first characterization of the role of the conserved DedA protein family in resistance to colistin and the first such study in any *Burkholderia* species.

Resistance to colistin in Gram-negative bacteria is usually conferred by activation of pathways that lead to the covalent modification of cell surface LPS, causing loss of electrostatic binding by the cationic antibiotic (Raetz et al., 2007; Olaitan et al., 2014). In *K. pneumoniae* and other species, these pathways are controlled by the PhoPQ and PmrAB two-component signaling pathways that lead to modification of lipid A with Ara4N and phosphoethanolamine (Olaitan et al., 2014; Poirel et al., 2017). While the energy requirements of the Ara4N pathway have not been characterized extensively, certain steps likely require the PMF (Figure 8). For example, it is known that transbilayer movement of the undecaprenyl-P-Ara4N from the cytoplasm to the periplasm require the EmrE-like transporters ArnEF (Yan et al., 2007) which are predicted to utilize the PMF. In addition, the undecaprenyl pyrophosphate phosphatase UppP/BacA displays amino acid similarity to the drug efflux pump MdfA and other secondary transporters and therefore may catalyze the transbilayer movement of undecaprenyl-P back to the cytoplasm in a PMF-dependent manner (El Ghachi et al., 2018; Workman et al., 2018). Therefore, tight control of PMF may be required for efficient modification of lipid A and resistance to colistin.

Extreme polymyxin resistance is a hallmark of *Burkholderia* species (Loutet and Valvano, 2011). While wild type enteric bacteria display resistance to low μg/ml concentrations of polymyxins (MIC 0.2–2 μg/ml) and resistant variants reach MIC up to 128 μg/ml^2^, *Burkholderia species* are often resistant to mg/ml concentrations and beyond (Burtnick and Woods, 1999; Thwaite et al., 2009; Loutet et al., 2011). In most resistant species, lipid A modification with aminoarabinose plays a major role, but it is possible that *Burkholderia* spp. employ other mechanisms including secreted proteases, utilization of efflux pumps, synthesis of putrescine, and hopanoid biosynthesis (Burtnick and Woods, 1999; Loutet and Valvano, 2011; Malott et al., 2012; El-Halfawy and Valvano, 2014). It is possible that the Δ*dbcA* mutation affects more than one resistance mechanism. The glycosyltransferase ArnT catalyzes the periplasmic modification of lipid A with Ara4N (Raetz et al., 2007; Petrou et al., 2016; Tavares-Carreon et al., 2016). While Ara4N synthesis is non-essential in most species and regulated by environmental cues (Raetz et al., 2007), ArnT is essential in *Burkholderia* spp. (Ortega et al., 2007; Hamad et al., 2012; Gislason et al., 2017) and appears to be required for LPS export (Hamad et al., 2012). For this reason, it is not possible to assess the colistin resistance of *B. thailandensis* in the complete absence of Ara4N modification without utilizing second site suppressors. The existence of a *B. thailandensis* mutant that inefficiently modifies lipid A with Ara4N will prove to be a valuable tool to study the role of the Ara4N pathway in LPS biogenesis in *Burkholderia* spp.

The DedA family of membrane proteins is widely distributed in nature, found in all kingdoms. Until recently, most of what is known about the family came from studies in bacteria due to pleiotropic phenotypes associated with null mutations. These phenotypes include defects in growth, cell division and sensitivity to alkaline pH, antibiotics and membrane penetrating dyes (Thompkins et al., 2008; Liang et al., 2010; Sikdar and Doerrler, 2010; Sikdar et al., 2013; Kumar and Doerrler, 2014, 2015). The correction of these phenotypes by growth in slightly acidic pH and the presence of membrane embedded charged amino acids suggest that members of the DedA family are proton-dependent transporters required for PMF maintenance (Sikdar et al., 2013; Kumar and Doerrler, 2014, 2015; Kumar et al., 2016). While there is no published structure of any DedA family protein, they do have an evolutionary relationship and, indeed may share structural similarity to proteins of the LeuT family of transporters (Khafizov et al., 2010; Keller et al., 2014). One of the earliest reports on a eukaryotic DedA family found the protein, called Tvp38, associated with tSNARE in Tlg-2 containing Golgi compartments in yeast (Inadome et al., 2007). Recent studies using CRISPR screening has demonstrated that a human DedA protein, known as TMEM41B, plays a role in autophagosome formation (Moretti et al., 2018; Morita et al., 2018; Shoemaker et al., 2019). While the eukaryotic proteins of the DedA family containing the so-called VTT domain (for VMP, TMEM41, Tvp38) (Morita et al., 2018) are distantly related to their bacterial counterparts, and a functional relationship has not been established, the presence of an absolutely conserved glycine residue suggests an evolutionary relationship (Tabara et al., 2019).

We have shown that a DedA family protein is required for intrinsic colistin resistance in *B. thailandensis*. Colistin sensitivity may be due to the requirement of the DedA family for proper PMF maintenance. We show that *B. thailandensis* Δ*dbcA* has lower membrane potential compared to the wild type. The colistin hypersensitivity seen with exposure to CCCP (Figure 7) also suggests that membrane potential is critical for colistin resistance in *B. thailandensis*. Membrane depolarization caused by CCCP has been shown to sensitize numerous Gram-negative species to colistin (Park and Ko, 2015; Ni et al., 2016; Osei Sekyere and Amoako, 2017; Baron and Rolain, 2018). Metabolically less active *Pseudomonas aeruginosa* biofilm cells were also more readily killed by colistin compared to metabolically active biofilm cells (Pamp et al., 2008). It is possible that the membrane depolarization of Δ*dbcA* has a negative impact on PMF dependent transporters that contribute directly or indirectly to colistin resistance. Efflux pump activity has recently been shown to be linked to lipid A modification with Ara4N and polymyxin resistance in *B. thailandensis* (Krishnamoorthy et al., 2019). Collectively, these results underscore the importance of maintenance of PMF in resistance to polymyxins as well as provide fresh insight into the roles of the widely distributed DedA family of membrane proteins.

## Data Availability Statement

All datasets generated for this study are included in the article/Supplementary Material.

## Author Contributions

WD, PP, and SK: conception or design of the study. PP, CB, CS, MD, CH, MT, and WD: the acquisition, analysis, or interpretation of the data. PP, MT, and WD: writing of the manuscript.

## Conflict of Interest

The authors declare that the research was conducted in the absence of any commercial or financial relationships that could be construed as a potential conflict of interest.

## Footnotes

1 http://tools.uwgenomics.org/tn_mutants/

2 https://mic.eucast.org/Eucast2/regShow.jsp?Id=39862
