# Supplementary material for: A DedA Family Membrane Protein Is Required for Burkholderia thailandensis Colistin Resistance
Source: Front Microbiol. 2019 Nov 5;10:2532. doi: 10.3389/fmicb.2019.02532 (PMC6849406; doi:10.3389/fmicb.2019.02532)
Supplement: Supplementary file 1 [file Presentation_1.pdf]

Supplementary Material

A DedA family membrane protein is required for *Burkholderia thailandensis* colistin resistance

Running title: DedA family required for colistin resistance

Pradip R. Panta[1†], Sujeet Kumar[1]*, Caitlin E. Billiot[1], Caroline F. Stafford[1], Martin V. Douglass[2,3], Carmen M. Herrera[2,3], M. Stephen Trent[2,3], and William T. Doerrler[1#]

Affiliations:
[1]Department of Biological Sciences, Louisiana State University, Baton Rouge, LA, USA
[2]Department of Infectious Diseases, University of Georgia, College of Veterinary Medicine, Athens, GA, USA
[3]Center for Vaccines and Immunology, University of Georgia, College of Veterinary Medicine, Athens, GA, USA

#Address correspondence to William T. Doerrler, wdoerr@lsu.edu

*Present address: The Ohio State University, Department of Microbiology, Columbus, OH, USA

Keywords: colistin; antibiotic resistance; lipopolysaccharide; membrane protein

Table S1. Bacterial strains and plasmids used in this study.

**Strains**	**Description**	**Source or Reference**
W3110	Wild Type *Escherichia coli*, F^{-}, λ^{-}, IN(*rrnD-rrnE)1,rph-1*	*E. coli* genetic stock center, Yale University
BC202	W3110 Δ*yqjA*::Tet^{R} Δ*yghB781::Kan^{R}*	(Thompkins et al., 2008)
BC203	W3110; Δ*yqjA*::Tet^{R}	(Thompkins et al., 2008)
BC204	W3110; Δ*yghB781::*Kan^{R}	(Thompkins et al., 2008)
BC202KS	W3110 Δ*yqjA*::Tet^{R} Δ*yghB781* (Kan-sensitive BC202)	This study
XL1 Blue	*recA*1 *endA1 gyrA96 thi-1 hsdR17 supE44 relA1 lac [F′ proAB lacIqZΔM15 Tn10* (Tet^{R})	Stratagene
E264	Wild-type *Burkholderia thailandensis*	(Brett et al., 1998)
Δ*dbcA*	E264 Δ*dbcA*::Tmp^{R}	This study
Δ*dbcA*::*FRT*	E264 Δ*dbcA::FRT*	This study
**Plasmids**		
pUC18T-mini-Tn7T-Tmp	Mini-Tn7T-based vector containing a trimethoprim resistance cassette; Tmp^{R} (GenBank accession no. DQ493875)	(Choi and Schweizer, 2006)
pBBR1MCS-2	Expression vector; RK2*mob lacZα E. coli lac* promoter, Kan^{R}	(Kovach et al., 1995)
pRP101	pBBR1MCS-2 expressing *dbcA*	This study

pRP102	pBBR1MCS-2 expressing Ec*yqjA*	This study
pRP103	pBBR1MCS-2 expressing Ec*yghB*	This study
pFlpTet	Rham-inducible *flp*, TS ori	(Garcia et al., 2013)
pSCrhaB2	Expression vector; ori$_{pBBR1}$*rhaR*, *rhaS*, *P*$_{rhaB}$Tmp^{R}*mob+*	Cardona and Valvano (2005)
pSC*dbcA*	pSCrhaB2 expressing *dbcA* with His$_6$ tag at C terminus	This study
pSC*dbcA*D67A	pSC*dbcA* with D79A point mutation in DbcA	This study
pSC*dbcA*E67A	pSC*dbcA* with E67A point mutation in DbcA	This study
pSC*dbcA*R161A	pSC*dbcA* with R161A point mutation in DbcA	This study
pSC*dbcA*R167A	pSC*dbcA* with R167A point mutation in Dbc	This study

Table S2. Oligonucleotide primers used in this study.

Primer name	Primer Sequence (5' → 3')
P1F	CAGATCGAGGAACAGCACGTAC
P1R	GATCCCCAATTCGAGCTCATGAAGCAAAAAAAGCAACGCTGTCA
P2F	ATGCATGAGCTCACTAGTGGAGAAGACGCAGGCGAACC
P2R	CTGTCGCACTATCTGGAATCCG
TmpF	AGCGTTGCTTTTTTTGCTTCATGAGCTCGAATTGGGGATCTTGAAGTA

TmpR	GC<u>GGTTCGCCTGCGTCTTCTCCACTAGTGAGCTCATGCATG</u>AT
Seq FW	TATATA*AAGCTT*CGATCTGGCTGATCAACTGGTC
Seq REV	ATATAATA*CTCGAG*GCAAGTGTATTGCGACACCAC
Confirm FW	ATGACAGCGTTGCTTTTTTTGC
Confirm REV	TCAGCGGTTCGCCTGCGTCTTC
M13FW	GTAAAACGACGGCCAGT
M13REV	CAGGAAACAGCTATGAC
FWdbcA	TATTAT*AAGCTT*ATGACAGCGTTGCTTTTTTTGC
REVdbcA	ATATAT*CTCGAG*TCAGCGGTTCGCCTGCGTCTTC
FWdbcAhis	ATATATCATATGGGATCTGATACAGCGTTGCTTTTTTTG
REVdbcAhis	GTGCCAAGCTTTTACTGCAGCTGTTGGCTGTGATGATGGTGGT GATGGCGGTTCGCCTGCGTCTTCTG
SeqpSCdbcAF	CATCATCACGTTCATCTTTCCCTG
SeqpSCdbcAR	GCAAATTCTGTTTTATCAGACCGC
D79AFW	TTCGCTGCTCTTCATCGCGGG
D79AREV	CGCCCGGCAGGAACGGGAAG
E67AFW	AACGGGGCTCGTGATCTTCCCG

E67AREV	CGCAGAAAACGATCAGGAACAGC
R161AFW	GCTCGCGGCCTTCATCCCGGTCGTGCG
R161AREV	GATGAAGGCCGCGAGCACGAGCGTCTTG
R167AFW	GGTCGTGGCCACGTTCGCGCCGTTCGTC
R167AREV	CGAACGTGGCCACGACCGGGATGAAGGC

Figure S1.

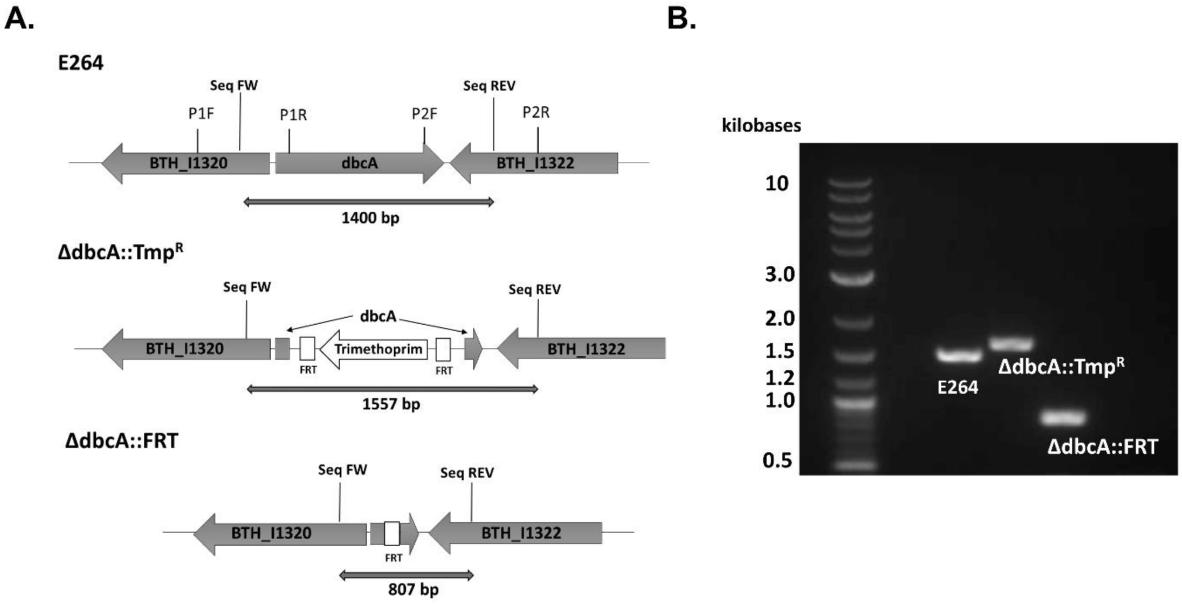

Fig. S1. Disruption of *B. thailandensis* E264 *bth_I1321* (*dbcA*). **A.** Diagram of *dbcA* locus along with adjacent open reading frames. *DbcA* is located between *BTH_I1320* (encoding mismatch repair protein MutL) and *BTH_I1322* (mechanosensitive ion channel YggB). The annealing sites of primers P1F, P1R, P2F, P2R, Seq FW, and Seq REV are shown. Genes are not drawn to scale. **B.** Ethidium bromide stained agarose gel with products of PCR reactions using primers Seq FW and Seq REV and genomic DNA from parent strain E264, trimethoprim resistant mutant strain (Δ*dbcA*::Tmp^R), and trimethoprim sensitive mutant strain (Δ*dbcA*::*FRT*). Presence and absence of the Tmp^R gene (*dhfrIIa*) was confirmed using DNA sequencing across the region with Seq FW and Seq REV primers.

References:

Brett, P.J., DeShazer, D., and Woods, D.E. (1998). Burkholderia thailandensis sp. nov., a Burkholderia pseudomallei-like species. *Int J Syst Bacteriol* 48 Pt 1**,** 317-320. doi: 10.1099/00207713-48-1-317.

Choi, K.H., and Schweizer, H.P. (2006). mini-Tn7 insertion in bacteria with single attTn7 sites: example Pseudomonas aeruginosa. *Nat Protoc* 1(1)**,** 153-161. doi: 10.1038/nprot.2006.24.

Kovach, M.E., Elzer, P.H., Hill, D.S., Robertson, G.T., Farris, M.A., Roop, R.M., 2nd, et al. (1995). Four new derivatives of the broad-host-range cloning vector pBBR1MCS, carrying different antibiotic-resistance cassettes. *Gene* 166(1)**,** 175-176.

Thompkins, K., Chattopadhyay, B., Xiao, Y., Henk, M.C., and Doerrler, W.T. (2008). Temperature sensitivity and cell division defects in an Escherichia coli strain with mutations in yghB and yqjA, encoding related and conserved inner membrane proteins. *J Bacteriol* 190(13)**,** 4489-4500. doi: 10.1128/JB.00414-08.
